# Role of Posttraumatic Stress Disorder Symptoms in Life Adaptation of Toxic Humidifier Disinfectant Survivors: A Multi-Group Analysis

**DOI:** 10.3390/healthcare14010083

**Published:** 2025-12-30

**Authors:** Yubin Chung, Min Joo Lee, Hun-Ju Lee, Soo-Young Kwon, Hye-Sil Ahn, Taksoo Kim, Sang Min Lee

**Affiliations:** 1 Counseling Program, Korea University, Seoul 02841, Republic of Korea; 2 Center for Public Health of Humidifier Disinfectant Survivors, Yonsei Unversity, Seoul 03722, Republic of Korea; 3 National Institute of Environmental Research, Incheon 22689, Republic of Korea

**Keywords:** social disaster, toxic humidifier disinfectant, PTSD symptoms, adaptation to life, multi-group analysis

## Abstract

**Background:** The Republic of Korean humidifier disinfectant disaster, involving toxic chemical exposure, constitutes a major social disaster causing severe trauma. While physical and psychological difficulties are documented, this study investigated the relationship between posttraumatic stress disorder (PTSD) symptoms and survivors’ daily life adaptation across children, adolescents, and adults, examining PTSD’s mediating role. **Methods:** The sample included 834 participants (417 exposed survivors and 417 unaffected individuals), divided into three age groups. PTSD symptoms and life adaptation were measured via self-reports. Multigroup Structural Equation Modeling (SEM) was utilized to test the indirect associations among exposure, PTSD symptoms, and life adaptation, and to examine age-group comparisons. **Results:** Survivors in all age groups reported higher PTSD symptoms and lower adaptive functioning compared to unaffected individuals, with the largest PTSD mean difference found in children and adults. Multigroup SEM confirmed that exposure positively impacted PTSD symptoms, and PTSD symptoms negatively impacted life adaptation across all ages. PTSD symptoms significantly mediated the exposure-life adaptation link in all groups. Critically, the direct effect of exposure on life adaptation was significant only in adults, indicating a full mediation via PTSD symptoms in children and adolescents. **Conclusions:** Exposure to toxic humidifier disinfectants is linked to life adaptation difficulties through elevated PTSD symptoms. These findings emphasize addressing trauma-related symptoms and suggest the utility of developmentally sensitive psychological interventions. Limitations include reliance on self- and parent-reported measures rather than clinical diagnoses, and the lack of control for external contextual factors (e.g., policy changes, media exposure).

## 1. Introduction

Humidifier disinfectant products containing chemicals that can cause fatal damage to the body through the lungs have been sold randomly in Korea for a long period of time [1,2]. From 1994 to 2011, approximately 9.98 million humidifier disinfectant products were sold to unspecified families in Korea [3,4].

These humidifier disinfectants contain toxic chemicals such as polyhexamethylene guanidine phosphate (PHMG-P), oligo 2-(2-ethoxy) ethoxy ethyl guanidine chloride (PGH), chloromethylisothiazolinone (CMIT), and methylisothiazolinone (MIT), which penetrate the human respiratory tract [5,6,7,8]. Given that humidifier disinfectants have been used for a long period of time, starting in 1994, for more than 15 years, the exact number of people exposed to this damage is unknown [3,4,9]. As of 30 November 2022, 7799 toxic humidifier disinfectant survivors had applied for compensation from the government. Of those 7799, 1797 survivors were already dead [10].

Toxic humidifier disinfectant damage has a significant effect on the human body, resulting in conditions such as respiratory failure and pulmonary fibrosis [11,12]. Several studies have shown that damage from humidifier disinfectants causes serious damage both physically [11] and psychologically [8,13,14]. In particular, Ko et al. [13] compared the mental health of 224 survivors and 228 norm samples between 2018 and 2021. The survivor group showed a higher presence of psychological symptoms, such as depression, withdrawal, anxiety, somatic complaints, thinking and attention problems, and aggression, than the general group. Furthermore, Lee et al. [14] analyzed the influence of psychological problems on life adaptation in 224 survivors of damage from humidifier disinfectants using canonical correlation analysis (CCA). The results showed that the higher the score of internalization (anxiety, depression, withdrawal, and body dissatisfaction) and externalization (aggressive behavior and rule violation), the more difficult relationships with spouses, partners, and friends became, and levels of maladjustment at work increased.

Damage caused by toxic disinfectants can largely be classified as a social disaster. While natural disasters such as earthquakes, forest fires, and tsunamis are one-off incidents, the social disaster of humidifier disinfectants had been occurring for over 15 years (1994–2011). This social disaster was also an unusual case in that entire households inhaled the disinfectants [3,15]. Considering this, we ask the following question: How serious is the psychological suffering of survivors of toxic disinfectants?

Disasters caused by toxic humidifier disinfectants are unprecedented and unique internationally. In addition, the harmful effects of this human-caused disaster on the mental health of survivors were more severe than the effects of natural disasters [16]; therefore, this rare type of social disaster must be scrutinized to understand the physical and psychological impacts on survivors and their family members with greater sensitivity. The humidifier disinfectant incident came to light in 2011, but the scope of compensation is unknown, and some survivors are still waiting to be compensated. On top of the direct negative impact due to chemical products, low social interest in the situation and delayed compensation have affected the lives of the survivors more destructively.

Han et al. [17] compared the number of prescriptions for psychotropic medications after the 2014 Sewol Ferry disaster (one of the major social disasters in Republic of Korea) in the Danwon district of Ansan city, a city in which many of the victims lived, to another city. The subjects who were prescribed the medications included survivors of the disaster as well as family members and acquaintances of the victims. The study showed that there was a significantly higher number of prescriptions for antidepressants in the Danwon district of Ansan City compared to other cities. It was implied that primary and secondary victims of social disasters had a higher risk of experiencing psychopathology after the disaster. Posttraumatic Stress Disorder (PTSD) occurs when a subject experiences a traumatic event and is a common psychopathology after disasters [16]. In the case of primary victims, the prevalence of PTSD is approximately 30%–40%, and children have a higher risk of developing PTSD than adults [16].

Subjects diagnosed with PTSD usually meet the conditions for other comorbid psychopathologies such as depression, anxiety, and panic disorder [18]. According to previous research, a high risk of developing PTSD after a disaster along with comorbid psychiatric disorders would impair the adjustment of survivors after a disaster. O’Donnell et al. [19] found that adjustment disorder has similar symptoms to those of PTSD. Difficulties with concentration, sleep problems, and irritability/anger, which are criteria for PTSD symptoms and intrusive memory, are also highly associated with adjustment disorder [19].

In the case of the humidifier disinfectant incident, the toddlers (1–3 years old) who were exposed to deleterious chemicals are still suffering from lung-related diseases. These lung diseases are difficult to treat, and treatment is needed for the rest of their lifetimes. Physical difficulties hinder survivors’ general functions and quality of life, but children’s chronic illnesses also negatively affect parents’ mental health. Cabizuca et al. [20] reported a high prevalence of PTSD among parents of children with chronic illnesses. This implies the importance of PTSD treatment plans for parents of children suffering from chronic illness.

Toxic damage is a social disaster, but it is an unprecedented and unique case in which chemicals are inhaled for a long time and cause damage to an entire family unit [3,15]. In addition, damage from toxic humidifier disinfectants not only poses a significant threat to physical health but also causes various psychological difficulties for survivors [13,14]. As discussed above, several studies have linked social catastrophe to PTSD symptoms [16,17,19,21,22].

While previous research has focused on various difficulties, there is a lack of empirical evidence from age-comparative analytic approaches concerning whether PTSD symptoms mediate the link between toxic exposure and life adaptation. Therefore, close examination of the PTSD symptoms of toxic humidifier disinfectant survivors is needed.

This study aimed to examine whether survivors at all developmental stages experience PTSD symptoms and how humidifier disinfectant exposure and PTSD symptoms affect adaptation to life. By applying a multigroup structural equation modeling approach across children, adolescents, and adults, this study uniquely contributes to the literature by clarifying developmental differences and similarities in the indirect association between toxic exposure, PTSD symptoms, and life adaptation. The research questions of this study are as follows: a) Does toxic humidifier disinfectant exposure affect PTSD symptoms in survivors? b) If toxic humidifier disinfectant exposure can affect survivors’ PTSD symptoms, how does it appear in each developmental stage, and how does it affect survivors’ life adaptation?

## 2. Methods

### 2.1. Participants and Procedures

The Institutional Review Board of Yonsei University in Republic of Korea granted ethical approval for this study (No. 7001988-202104-HR-1178-02l). Data have been collected starting in 2018, when the National Environmental Research Institute (NIER) launched a government-funded program to monitor survivors’ mental health. In this study, data from the first year (2018) to the fourth year (2021) were used for the analysis. People officially identified by the government as toxic humidifier disinfectant survivors are eligible for mental health care services under the program. Thus, the participants in this study were those who voluntarily enrolled in the program to receive counseling and psychological services. A total of 417 survivors participated in the study and completed the survey prior to the counseling session. Normative data were provided by HUNO, the organizer of the Achenbach System of Empirically Based Assessment (ASEBA) in Republic of Korea. Age-specific normative samples for group comparisons were then randomly selected from an equal number of survivors (*n* = 417).

### 2.2. Measures

This study utilized the ASEBA, which offers a systematic and comprehensive assessment of behavioral problems and adaptive functioning [23]. ASEBA is a standardized broad-coverage evaluation tool that is widely utilized in both clinical and scientific settings. The strength of ASEBA is that it can be used for subjects of all ages, from children to the elderly, and it provides comparable scores across different age groups [24]. The survivors responded to ASEBA according to their age groups. For elementary school students, parents or primary caregivers responded to Child Behavior Checklist (CBCL). Secondary students and adults self-responded to Youth Self Report (YSR) and Adult Self Report (ASR), respectively. In this study, the Korean versions of the CBCL and YSR validated by Oh and Kim [25] and ASR validated by Kim et al. [26] were used.

#### 2.2.1. PTSD Symptoms

To assess the level of PTSD-related symptoms in participants, items from the ASEBA system were utilized, including the CBCL, YSR, and ASR. Although these measures do not include a subscale that directly assesses PTSD, they contain items related to posttraumatic stress responses within the depression, anxiety, and internalizing problem subscales. Based on previous studies, these items were combined to construct indicators of trauma-related symptoms, which were used to evaluate the level of PTSD-related symptoms. Wolfe et al. [27] proposed these items to identify trauma symptoms, which Wolfe [28] later updated. Previous studies have confirmed the PTSD items of ASEBA as a useful screening tool for the detection of PTSD [29,30]. Fourteen items rated from 0 (not true) to 2 (very true or often true) were used. Sample items include “Trouble Sleeping,” “Cannot get his/her mind off certain thoughts, obsessions,” and “Nervous, high-strung, or tense.” Sim et al. [31] and Milot et al. [32] provided evidence of the construct validity of the PTSD subscale (i.e., convergent validity).

#### 2.2.2. Adaptive Functioning

The adaptive functioning subscale was used to assess participants’ adaptive functioning. For adult participants, the adaptive functioning subscale consisted friends, spouse/partner, family, job, and education. For children and adolescent participants, the adaptive functioning subscale comprises sociality and academic performance. We used standardized scores for comparison while also reflecting developmental differences in functional domains. As a result, the total score of the adaptive functioning subscale measured in each group (children, adolescents, and adults) was converted into a *T* score (i.e., standardized score) and used for analysis in this study. A lower score indicates a lower level of adaptation and vice versa. The following are the reliabilities (Cronbach’s α) of the scale according to age and humidifier disinfectant exposure: Children–Norm = 0.60, Children–Survivor = 0.65, Adolescent–Norm = 0.47, Adolescent–Survivor = 0.56, Adult–Norm = 0.76, and Adult–Survivor = 0.70.

### 2.3. Data Analysis

This study examined the mediating role of PTSD symptoms in the association between exposure to toxic disinfectants and adaptive functioning within a cross-sectional design, using data collected at a single time point for each participant. Those who were exposed to toxic disinfectants were coded as 1 as survivors and those who had never been exposed to toxic disinfectants were coded as 0 as the norm group. In addition, we examined whether the relationship between the variables showed a consistent pattern across the three age groups: adults, middle/high school students (i.e., adolescents), and elementary school students and younger (i.e., children). As for age group comparisons, we tested the hypothesis using multi-group structural equation modeling (SEM). The sequence of data analysis performed in this study was as follows:

Descriptive statistics, correlations, and reliability were analyzed using IBM SPSS version 21.0 (IBM Corp., Armonk, NY, USA). Next, we performed a series of analyses with multi-group SEM using Amos version 21.0. In Amos, missing values are handled automatically using the full-information maximum likelihood (FIML). Initially, we examined the measurement model to ensure that the observed variable adequately measured the corresponding latent variables. The Comparative Fit Index (CFI), Tucker–Lewis Index (TLI), and Root Mean Square Error of Approximation (RMSEA) were used to evaluate the fitness of the model. CFI and TLI results greater than 0.95 were interpreted as a very good model fit, while an RMSEA of less than 0.05 was interpreted as a very good model fit [33]. We then tested the measurement invariance, a primary requirement for multi-group SEM, to examine the measurement consistency of the variables across groups. To test measurement invariance, factor loadings were constrained to be equal across age groups. Measurement invariance was considered satisfactory if there was no statistical change in CFI and RMSEA between the constrained and unconstrained models [34]. As suggested by Chen [34], since the number of samples differed between groups and was less than 300, invariance was interpreted as satisfactory when ΔCFI < −0.005 and ΔRMSEA < 0.01. The final model and path coefficients between variables were identified accordingly. Finally, the significance of the mediating effect of PTSD symptoms on the relationship between toxic disinfectant exposure and adaptive functioning was examined using the bootstrap method. Bias-corrected 95% confidence intervals for the estimates of indirect effects were calculated using 5000 bootstrap samples. If the upper and lower limits of the 95% confidence interval did not contain zero, the estimate of the indirect effect was interpreted as significant [35].

## 3. Results

### 3.1. Descriptive Statistics and Correlations

The means, standard deviations, and correlations are presented in Table 1. The average PTSD symptom score of the survivors was higher than that of the norm group in all three age groups. Children and adults showed a very large mean difference, whereas adolescents showed a moderate mean difference. In contrast, the average score of adaptive functioning of the survivors was lower than that of the norm group in all three age groups. Overall, the level of adaptive functioning showed moderate mean differences between the survivor and norm groups. Correlation analysis revealed that PTSD symptoms were significantly correlated with adaptive functioning (*r_s_* = −0.37–0.49), except in adolescents in the norm group (*r* = −0.22, *p* = 0.08).

### 3.2. Multigroup SEM

**Figure 1:**
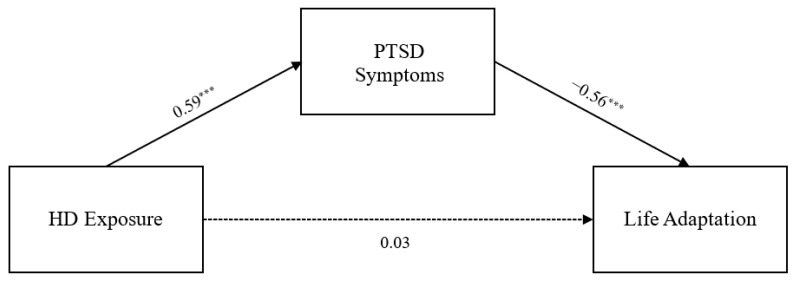
Research Model for Children. *Note.* HD = humidifier disinfectant. Standardized path coefficients correspond to children. *** *p* < 0.001. Solid lines indicate statistically significant relationships between variables, and dashed lines indicate non-significant relationships.

**Figure 2:**
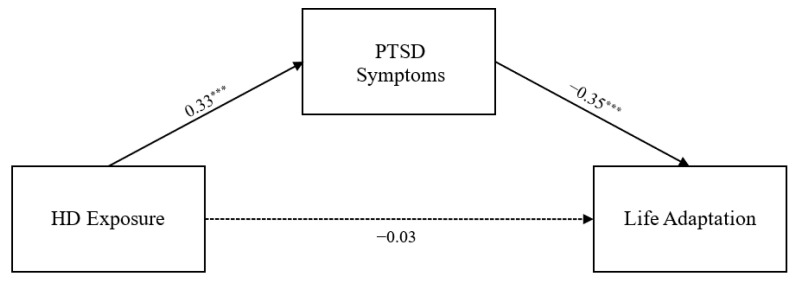
Research Model for Adolescents. *Note.* HD = humidifier disinfectant. Standardized path coefficients correspond to adolescents. *** *p* < 0.001. Solid lines indicate statistically significant relationships between variables, and dashed lines indicate non-significant relationships.

**Figure 3:**
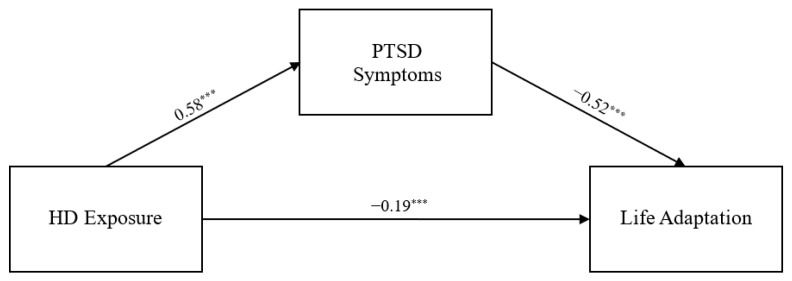
Research Model for Adults. *Note.* HD = humidifier disinfectant. Standardized path coefficients correspond to adults. *** *p* < 0.001. Solid lines indicate statistically significant relationships between variables.

The model fit indices of the measurement model were acceptable ($\chi^{2}$ = 7.396, *df* = 4, *p* = 0.116, CFI = 0.998, TLI = 0.995, RMSEA = 0.032). All criteria for the goodness-of-fit indices were satisfied. Next, we examined whether the variables were measured identically in the three age groups, and confirmed the final model using measurement invariance tests. In this study, the fit indices of both the configural invariance model ($\chi^{2}$ = 14.705, *df* = 12, *p* = 0.258, CFI = 0.999, TLI = 0.996, RMSEA = 0.016) and the measurement invariance model ($\chi^{2}$ = 24.527, *df* = 16, *p* = 0.079, CFI = 0.995, TLI = 0.991, RMSEA = 0.025) were found to be good. In addition, there were no significant differences in ΔCFI (<−0.005) and ΔRMSEA (<0.01) between the models, confirming the equivalence of factor loadings across age groups. Therefore, the measurement invariance model was adopted as the final model. The path coefficients of the final model are presented in Table 2 and Figure 1, Figure 2 and Figure 3. As expected, toxic disinfectant exposure had a significant positive impact on PTSD symptoms in all three age groups (β_s_ = 0.33–0.59, *p* < 0.001), and PTSD symptoms had a significant negative impact on adaptive functioning in all three age groups (β_s_ = 0.35–0.56, *p* < 0.001). However, the direct effect of toxic disinfectant exposure on adaptive functioning was still significant in adults (β = −0.19, *p* < 0.001) but was no longer significant in children and adolescents.

### 3.3. Significance of Indirect Effects of PTSD Symptoms

As shown in Table 3, the 95% confidence interval for the mediating effect of PTSD symptoms on the relationship between toxic disinfectant exposure and adaptive functioning did not include zero in any of the three groups. Therefore, in this study, the indirect effect of toxic disinfectant exposure on adaptive functioning via PTSD symptoms was statistically significant in all of the age groups. Moreover, in the case of children and adolescents, the direct path of exposure to toxic disinfectants on adaptive functioning was not significant, indicating that it had a full mediation effect.

## 4. Discussion

In this study, we examined the effect of humidifier disinfectant exposure on the adaptive functioning of survivors in a group including children, adolescents, and adults. We also examined the association between PTSD symptoms and life adaptation in survivors. In this study, we utilized ASEBA [24,36] as a tool for assessing PTSD symptoms of survivors in all three age groups. The ASEBA scale has been employed in various studies to identify psychopathology [8,36,37]. As one of the major psychological assessments in psychiatric and counseling settings in Republic of Korea [38], the ASEBA sub-scales for PTSD symptoms and adaptive functioning are expected to provide crucial details related to survivors’ mental health.

The average score on the PTSD scale of survivors of humidifier disinfectants is higher than that of the general population in all groups of children, adolescents, and adults. In particular, the PTSD scores of survivors among children and adults were much higher than those of the adolescents. The PTSD subscale of ASEBA divides the criteria into three groups: clinical (T > 70), borderline (T = 65–69), and normal (T < 64). The average scores for the children’s group were 71.85, and the average scores of the adult group were 65.55. Considering that a T score of 70 is in the top 98% of percentile scores, the results indicate that the child survivors were suffering from severe post-traumatic stress disorder, and that the adult group had borderline post-traumatic stress disorder.

Over a decade has passed since the government announced that the unexplained lung diseases that were occurring were caused by humidifier disinfectants. However, survivors still experience PTSD symptoms at a high rate [29,39].

Previous research on the mental health of humidifier disinfectant survivors has found that survivors experience psychological symptoms such as somatic complaints, thinking and attention problems, and aggression compared to the general population [8,13]. Furthermore, humidifier disinfectant survivors with severe psychological symptoms have significant difficulties adjusting to life [14]. To date, we have not found any research on PTSD in humidifier disinfectant survivors. Various studies have shown that survivors of natural disasters, war veterans, and social disasters report having PTSD after experiencing disasters [40]. In addition, several studies have reported that a significant number of survivors suffer from PTSD in the long term after experiencing a disaster [21,41]. Even today, the government and companies are shifting the blame for humidifier disinfectant disasters onto one another, while still failing to provide adequate compensation. This string of incidents in the humidifier disinfectant case creates an environment that can lead to even more fatal PTSD in survivors.

In systematic reviews [22,42] that examined PTSD prevalence based on disaster characteristics, it was found that human-made or technological disasters may have different and more severe effects than natural disasters. This can be interpreted to mean that acts of brutality from fellow humans betray people’s basic trust, affecting them even more than natural disasters. The humidifier-disinfectant disaster incident may be understood as a compound disaster that could have contributed to the erosion of public trust and aspects of the social security system. The results of this study suggest that survivors exposed to these events may experience significant mental anguish.

According to several studies [43,44], most survivors face practical difficulties with adapting to life. The results of this study were consistent with those of previous studies. The mean score of the adaptation scale for humidifier survivors was much lower than that of the norm group for all age groups. This suggests that humidifier disinfectant survivors also have trouble adjusting to everyday life. The correlation between the PTSD scale score and the adaptation scale was also significantly negative in all groups. This means that the more severe the degree of PTSD, the more difficult it is to adapt to various areas of life. The findings of this study are worth noting because PTSD goes beyond subjectively experienced psychological distress and is closely associated with adaptation in various areas of life.

Another important finding of this study is that the pathways through which disaster exposure affects life adaptation differ across age groups. The SEM results showed that, in the adult group, exposure to humidifier disinfectants had both a direct effect on life adaptation and an indirect effect mediated by PTSD symptoms. In contrast, among children and adolescents, no direct effect of exposure on life adaptation was observed; rather, the impact of exposure on life adaptation emerged only through PTSD symptoms.

These age-specific patterns can be understood from a developmental perspective. Children and adolescents are more vulnerable to trauma-related symptoms due to still-developing capacities for emotion regulation and meaning-making, and their daily functioning is more strongly embedded in external environments such as family and school contexts [45]. As a result, disaster exposure in younger populations may primarily manifest as psychological symptoms, which subsequently influence life adaptation. In contrast, adults tend to integrate traumatic experiences into broader role-based life scenes, including work, family responsibilities, and long-term life planning, making both direct and symptom-mediated pathways to life adaptation more salient [46].

Several studies suggested that psychological difficulties experienced by survivors of such incidents vary by age group [43,47]. The intervention method, timing, and preemptive intervention before development into a serious psychopathic disease, considering the characteristics of each age group, could provide efficient systematic help for the survivors’ development and subsequent adaptation to life. A strength of this study is that it examined how catastrophic events may be associated with PTSD and adjustment to life across age groups from children to adults.

This study explored the differences and similarities in patterns of PTSD symptoms and life adjustment by age group in survivors of humidifier disinfectant use. A correct understanding of the psychological distress experienced by survivors through objective and comprehensive measurements is considered a prerequisite for healing and recovery. The results of this study may provide a basis for the design of age-appropriate interventions for survivors.

## 5. Limitations

Several limitations of this study should be noted. First, the study participants were defined as individuals who voluntarily enrolled in a counseling and psychological support program following exposure to humidifier disinfectants. As such, the sample may not be representative of all affected individuals, as those experiencing greater psychological distress or higher motivation to seek help may have been more likely to participate. This self-selection may have introduced selection bias, potentially limiting the generalizability of the findings to the broader population of survivors.

Second, in this study, DSM criteria are used to diagnose PTSD based on individual interviews with clinicians and patients, which limits diagnoses in the case of a large-scale disaster situation, in which there are a large number of survivors. In cases such as the humidifier disinfectant disaster, with many survivors, the self-reported checklist was considered a more appropriate tool for screening and assessing PTSD. Therefore, in this study, the ASEBA was adopted as a tool for the assessment of PTSD symptoms in all three age groups. The ASEBA scale has been employed in various studies to identify psychopathology and is a proven and relevant tool for diagnosis in psychiatric settings [29,31]. Though its results can reveal PTSD symptoms experienced by the survivors, they cannot be clinically diagnosed with PTSD based on ASEBA results alone. Additional in-depth interviews with clinicians and psychological tests are required. After screening survivors with PTSD symptoms using ASEBA results, PTSD diagnoses should be made through additional tests for the risk group.

Third, during the data collection period, external factors such as changes in compensation policies, media re-reporting of the disaster, and the COVID-19 pandemic may have influenced participants’ psychological symptoms. However, individual-level data on these factors were not available and thus could not be controlled for analysis. Future longitudinal studies that account for these contextual influences are needed to clarify the temporal dynamics of PTSD symptoms and adaptive functioning among disaster survivors.

Lastly, although the use of parent reports for children and self-reports for adolescents and adults is unavoidable in a cohort study (or age-based studies), reporting bias causes disagreement about individuals’ psychological states. Because the CBCL is a parent account of a child’s behavior, it comes with the limitations inherent in such instruments, such as reporting bias or difficulty in inferring a child’s internal state.

## 6. Conclusions

Based on the findings of this study, it can be concluded that exposure to humidifier disinfectants significantly affects the psychopathology of children, adolescents, and adults. Compared to the general population in all age groups, survivors of humidifier disinfectant exposure had higher average scores on the PTSD scale, indicating long-term psychological difficulties related to PTSD. The survivors also had lower scores on the life adaptation scale of ASEBA than the normative group, suggesting that they are facing ongoing psychological challenges. A key strength of this study lies in its inclusion of survivors across multiple developmental stages and its use of multigroup analytic approaches, which allowed for a comprehensive examination of age-related similarities and differences in the associations among exposure, PTSD symptoms, and life adaptation.

However, this study relied on self- and parent-reported measures rather than clinical diagnoses, and potential contextual influences could not be fully controlled, which may limit causal interpretation of the findings.

Despite these limitations, the present findings provide important implications for future research and practice, suggesting the need for longitudinal and multimethod studies and for the development of developmentally sensitive screening and intervention strategies to support the long-term psychological recovery of humidifier disinfectant survivors. Interventions for survivors, including effective identification and treatment, are vital for addressing these difficulties and reducing residual psychopathology.

## Figures and Tables

**Table 1:** Descriptive statistics and correlations.

Group	HD Exposure	Variables				PTSD-LA
		PTSD Symptoms		Life Adaptation		
		*M*	*SD*	*M*	*SD*	Correlation
Children	Norm	50.00	10.00	50.00	9.92	−0.49 ***
	Survivors	71.85	21.02	43.31	11.19	−0.42 ***
Adolescents	Norm	50.00	10.00	50.00	9.50	−0.22
	Survivors	56.80	11.13	46.62	14.05	−0.37 **
Adults	Norm	50.00	10.00	50.35	5.99	−0.41 ***
	Survivors	65.55	13.91	42.86	7.24	−0.48 ***

*Note.* HD = humidifier disinfectant, LA = life adaptation. ** *p* < 0.01, *** *p* < 0.001.

**Table 2:** Regression path coefficients of the final model.

Path	Group	B	β	SE	*t*
HD Exposure → PTSD symptoms (*a*)	Children	0.41	0.59	0.04	10.46 ***
	Adolescents	0.23	0.33	0.06	3.60 ***
	Adults	0.47	0.58	0.04	13.08 ***
PTSD symptoms → Adaptive Functioning (*b*)	Children	−17.91	−0.56	2.45	−7.33 ***
	Adolescents	−11.93	−0.35	3.30	−3.61 ***
	Adults	−9.82	−0.52	0.96	−10.23 ***
HD Exposure → Life Adaptation (*c’*)	Children	0.59	0.03	1.55	0.38
	Adolescents	−0.63	−0.03	2.16	−0.29
	Adults	−2.92	−0.19	0.71	−4.09 ***

*Note.* HD = humidifier disinfectant; B = unstandardized coefficient; *β* = standardized coefficient; SE = standard error. *** *p* < 0.001. The arrows indicate statistically significant relationships between variables.

**Table 3:** Significance of indirect effects of PTSD symptoms.

Path			95% Confidence Intervals	
HD Exposure → PTSD symptoms → Life Adaptation	B	SE	LL	UL
Children	−7.30	1.32	−10.12	−4.88
Adolescents	−2.74	1.22	−5.89	−0.98
Adults	−4.57	0.59	−5.81	−3.51

*Note.* Bias-corrected 95% confidence intervals (CIs) are presented. HD = humidifier disinfectant; B = unstandardized coefficient; SE = standard error; LL = lower limit; UL = upper limit. The arrows indicate statistically significant relationships between variables.

## Data Availability

The data presented in this study are available on request from the corresponding authors. The data are not publicly available due to privacy and ethical restrictions.
